# Annotations

**Published:** 1908-05-16

**Authors:** 


### Annotations.

### Liability for a Consultant's Fee.

There has lately been decided in the Supreme Court of the Cape Colony a somewhat unusual action between two medical men over a question of fees. Briefly the dispute turned on the point whether a consultant should look for his fee to the practitioner by whom he is called in or to the patient. It appears that in many districts the former custom prevails, the attending practitioner recovering it subsequently from the patient. But it is very easy to see the disadvantages of such a system. Thus an insistent but impecunious patient may unnecessarily demand a consultation, and the practitioner, who may be somewhat doubtful about his own fees, will naturally feel somewhat sore if required to pay from his own pocket those of a consultant. Moreover, the usual practice of paying cash for a consultation works well in several ways. The patient knows what the proceeding costs him, and has not to disentangle this from the account of his attending practitioner, whose fees he may have occasion to dispute. The consultant is not likely to suffer if he or the practitioner makes it clear to patients that the transaction is to be on a business footing, and those who might cherish ideas of imposing on the medical profession will then probably abandon them. There are, no doubt, considerations which weigh in favour of the opposite plan; perhaps local conditions of colonial life with which we in this country are necessarily unfamiliar have led to its adoption. One such consideration is possibly the fact that, as the consultant is very often a neighbouring practitioner of the same or the nearest town, it is undesirable in the interests of professional harmony that the patient of the consulter should pay the consultant direct.

### Territorial General Hospitals.

Amid the chaos which characterised the organisation, or lack of organisation, of the Auxiliary Forces until the origination of the Territorial scheme, few departments were more prominent than the medical. Had the Volunteers been mobilised for the defence of our shores, the first result would have been a complete breakdown of the system for coping with sickness, quite apart from the question of the fate of the wounded after an action. It was accordingly with great interest that all members of the profession who are or have been connected with the Auxiliary Forces regarded the comprehensive scheme for remedying the old state of things which was put forward by the War Office under the energetic sponsorship of the Director-General. One of the most novel features of this scheme is the provision of general hospitals in different parts of the country, to be officered mainly by surgeons and physicians drawn from the ranks of the consultants, and by preference on the staff of large hospitals in teaching centres. The draft regulations contained brief outlines from which it appeared that these institutions are to exist on paper only, as far as rank and file are concerned, until the mobilisation of the Territorials; and that the officers are not to be called on for drills or camp attendance, as are those attached to individual units or field ambulances. In fact, the general hospitals under this scheme constitute a third line of medical organisation for the home defence forces. It is, therefore, gratifying to observe that the scheme is maturing steadily. Four general hospitals are to be allotted to London, two to Glasgow, and one each to Edinburgh, Birmingham, Plymouth, Manchester, Sheffield, Aberdeen, Newcastle-on-Tyne, Liverpool, Leeds, Lincoln, Leicester, Oxford, Cambridge, Cardiff, Bristol, Portsmouth, and Brighton. The British Red Cross Society is to participate in the provision and equipment of these hospitals, and if the Treasury consents to due financial support there ought to be no difficulty in obtaining a fine *personnel*. The rock on which the scheme may split is the non-training clause: marquees, stretchers, beds, and so forth, never used and never unpacked, will deteriorate whilst in store to such an extent as to prove unserviceable in emergency unless very carefully inspected from time to time. The establishment of general hospitals on this skeleton system will be a delusion unless the equipment is issued and maintained at a very high pitch of excellence: dressers and nurses can be improvised in time of national peril from our urban hospitals, but horses and waggons, tents, sterilisers, hot-water bottles, and all the thousand and one requirements of a military hospital cannot be thus charmed into existence; they must be fully and thoroughly considered beforehand.

### Hospital or Infirmary?

In London the word "Infirmary" is applied almost exclusively to Poor-law Infirmaries, and, now that Christ's Hospital has moved into the country, the word "Hospital" is reserved for institutions endowed or maintained by charity for the relief of bodily or mental disease. In a considerable number of the great Scottish and provincial cities, on the other hand, the principal hospitals retain the name of "Infirmary," in many cases with the honourable prefix of "Royal." Of the two words—although "infirmary" is perhaps the more precise, "hospital" has yet the more dignified etymology and associations. The fact that the Poor-law authorities have annexed the other, should not of course affect its meaning. But of late years, especially in the southern counties of England, a prejudice has arisen in the lay mind against the retention of the title of "infirmary" for institutions which correspond to the London idea of hospitals, and this has led in several instances to the substitution of the one name for the other. We now learn that the Hertford General Infirmary proposes to follow this course. The authorities, while recognising that a feeling of sentiment naturally clings to a name which long use has made familiar, are of opinion that the proposed change is desirable in the best interests of the Infirmary. On account of the co-existence of a local Poor-law infirmary there is some confusion in the minds of the inhabitants as to what is meant when the word "infirmary" is used; while the staff of the General Infirmary consider that the change of name will improve its status and their's, in the eyes of the medical world. We can see no possible objection to re-christening the institution in accordance with modern ideas.

